# Does it matter who organises your health care?

**DOI:** 10.5334/ijic.1598

**Published:** 2015-05-14

**Authors:** Paresh Dawda, Ian S McRae, Laurann Yen, Md Mofizul Islam, Nasser Bagheri, Tanisha Jowsey, Michelle Banfield, Anne Parkinson

**Affiliations:** Australian Primary Health Care Research Institute, Australian National University, Research School of Population Health, College of Medicine, Biology and the Environment, The Australian National University, Canberra, Australia; ACT Medicare Local, ACT 2601 and Ochre Health, Calwell ACT 2905, Australia; Australian Primary Health Care Research Institute, Australian National University, Research School of Population Health, College of Medicine, Biology and the Environment, The Australian National University, Canberra, Australia; Australian Primary Health Care Research Institute, Australian National University, Research School of Population Health, College of Medicine, Biology and the Environment, The Australian National University, Canberra ACT 2601, Australia; Australian Primary Health Care Research Institute, Australian National University, Research School of Population Health, College of Medicine, Biology and the Environment, The Australian National University, Canberra ACT 2601, Australia; Australian Primary Health Care Research Institute, Australian National University, Research School of Population Health, College of Medicine, Biology and the Environment, The Australian National University, Canberra ACT 2601, Australia; Australian Primary Health Care Research Institute, Australian National University, Research School of Population Health, College of Medicine, Biology and the Environment, The Australian National University, Canberra ACT 2601, Australia; Australian Primary Health Care Research Institute, Australian National University, Research School of Population Health, College of Medicine, Biology and the Environment, The Australian National University, Canberra ACT 2601, Australia; National Institute for Mental Health Research, Australian National University, Research School of Population Health, College of Medicine, Biology and the Environment, The Australian National University, Canberra ACT 02601, Australia; Australian Primary Health Care Research Institute, Australian National University, Research School of Population Health, College of Medicine, Biology and the Environment, The Australian National University, Canberra ACT 2601, Australia

**Keywords:** coordination, chronic disease, long-term conditions

## Abstract

**Background:**

As the prevalence of long-term and multimorbid conditions is increasing, patients increasingly require consultations with multiple health care professionals and coordination of their care needs.

**Methods:**

This study is based on a 2011 survey of older Australians which draws on sub-populations of people with diabetes aged 50 years or over, people with chronic obstructive pulmonary disease, and members of Nationals Seniors Australia. We develop a composite coordination measure and examine differences in the measure with different care coordination indicators using both descriptive and regression methods. Three categories of respondent-perceived care organisers are used: health care professionals; “no one”; and patients, their partner, relative or friend.

**Results:**

Of the 2,540 survey respondents (an overall response rate of 24%), 1,865 provided information on who organised their health care, and composite coordination measures were calculated for 1,614. Multivariate analysis showed the composite score was highest where a health care professional coordinated care, followed by care organised by self or a carer, and then the group reporting no organiser.

**Conclusion:**

In moving towards care coordination there are opportunities to improve the care coordination process itself, and the key enablers to improving care coordination appear to be the availability and communication of clinical information and the role of the clinical team.

## Introduction

One of the characteristics of the changing demographics of populations across the world is the increasing burden that long-term conditions (LTCs) place on all health systems. The prevalence of LTCs is increasing, as is the number of concurrent conditions suffered by an individual [1, 2]. In our complex, multipart health systems, the patient journey increasingly requires multiple consultations with multiple providers of care including General Practitioners (GPs), Specialists, Nurses and Allied Health Care providers. In addition, the accompanying social problems necessitate communication across the health and social interface.

Coordination of care is an essential component for the delivery of high-quality primary health care that offers benefits for both patient care and health outcomes [3–6]. This move towards coordinated team-based health care is an attempt to better address the needs of an ageing population and growing incidence of people with chronic and complex conditions where care is required across multiple care settings, often in different locations [7]. The definition of coordinated care is variable and has changed over time. McDonald et al. [8] define care coordination as:

The deliberate organization of patient care activities between two or more participants (including the patient) involved in a patient's care to facilitate the appropriate delivery of health care services. (p. 41)

Coordination of care relates to the interrelationship between and within multiple levels of the health care system including micro level (patients and service providers), meso level (health service organisations) and macro level (health services) [7]. Key to understanding coordination of care is to take a broad view and recognise its association with continuity of care, integration of care and patient-centred care [9].

Coordination of care has been identified as a national priority area in the primary health care strategy of Australia, and elsewhere [10–12]. Many models of chronic disease care, such as the Wagner model and Flinders model incorporate the need to support patients in managing their own care and the need for an “activated patient” to deliver quality clinical and functional outcomes [13, 14]. These ideas are now firmly incorporated in policy statements such as Australia's National Chronic Disease Strategy [15].

Most measures of coordination deal with patient perception of how well their care is coordinated. However, the field lacks a way of identifying actual coordination. In this study we develop such a measure based on activities related to coordination, such as availability of information, duplication of testing and measures of medication management. We then explore who it is that older Australians consider coordinates their health care, and observe whether there are any differences in coordination measures with different care coordinators. While we anticipate that where care is pro-actively managed this coordination will be reflected in the outcomes measured, a priori we do not know if access to a professional coordinator is materially more effective than careful self-coordination.

## Methods and data

The study is based on a 2011 survey of three sub-populations of older Australians, including National Seniors Australia (NSA), an organisation of Australians aged 50 and over with 285,000 members from which a sample of 5,000 people was drawn. NSA broadly represents the older Australian community. The second sub-population is the National Diabetes Services Scheme (NDSS), a government funded service, which provides subsidies for diabetes materials with 280,000 of its registrants aged over 50 years (sample size 2,500). The third sub-population is the Lung Foundation Australia (LFA), a member organisation which supports research into lung conditions and provides member support (all 3,109 members with chronic obstructive pulmonary disease (COPD) or who supported a person with COPD were included in the sample). Almost all people in this group were aged over 50 years.

Stratified random samples were drawn from each of the sub-populations using age/sex/state/rurality strata, with some oversampling of more rural areas to ensure adequate rural samples. For more detail, see Jowsey et al. [16].

The development of the questionnaire has been reported elsewhere [16]. The questionnaire has three sections (a) Demographics (b) Health, Coordination and Time Use for managing the respondent's own health and (c) Carer Time Use for managing the caree's health. Part (b) of the survey comprises four sections (1) Health (2) Health Service Use (3) Working Together and (4) Time Use.

The questionnaire was based on that previously tested and used by McRae et al. [17] and incorporated questions which focused on events which had actually occurred related to care coordination rather than on perceptions of coordination. These questions were drawn or adapted from existing surveys including, the Assessment of Chronic Illness Care (ACIC) and Patient Assessment of Chronic Illness Care (PACIC) [18, 19]. The questionnaire was piloted with 18 members of a local health service consumer network, and the revised survey re-tested with 28 older Australians who had taken part in an earlier survey and indicated their willingness to participate in further research.

### Development of a composite index of coordination

The questions in the “Working Together” section relate to coordination of care including the availability of information to health professionals, the use of written care plans, and using management of medication changes as a marker for coordination of care.

The composite coordination index was computed using a set of eight questions that were drawn together by an expert committee after completion of the survey. The questions used were identified as those which addressed the main communication and management domains of care coordination (including care planning) addressed by the survey. The questions used are given in Table 1 together with the weights applied to each of the possible answers. The weights used involved transforming each individual item to a 0–100 scale with higher scores indicating more favourable experience (coordination) as shown in Table 1. For most questions (apart from missing values) there were only scores of 0 or 100, however two questions had intervening values (e.g. “sometimes”) and these values were assumed to be uniformly spread across 0 to 100 meaning scores of 33.3 and 66.7 were applied as per Table 1. Since items 3 and 4 relating to care plans basically ask the same question but refer to different providers, they were combined to develop one item, bringing the total number of questions to seven. Taking a conservative approach, a response of “Don't know” was treated as “No”, and “Not applicable” as “Missing”.

The composite coordination index was computed for each respondent based on the average of responses to all items mentioned above, using the “half-scale rule” [20, 21]. The half-scale rule calculates scale scores only for respondents who answer at least 50% of the items in the scale – those who answer less than 50% of questions are excluded from the analysis. For those included the overall score is the average value over the available questions, which is equivalent to imputing the mean value of the available items for any missing items.

The three categories of care organisation were derived from the survey question “Who is the *main* person who organises your health care?” which provided response categories of “no-one”, “specialist”, “practice nurse”, “myself”, “my partner/spouse”, “relative”, “friend”, “GP”, “Other”. The simplified categories were those who stated that “No-one” organises their care, those whose care is organised by a health care professional (Specialist, Practice Nurse, GP) and those whose care is organised by themselves or by a partner, relative or friend.

Most of the descriptive analysis is undertaken within the sub-samples to establish the basic relationships. Totals across the sub-samples are used for simplicity when exploring the relationship between the main organiser and individual questions. The central analysis of the relationship between coordination (measured by the composite coordination index) and the main organiser with allowance for sub-sample, complexity of conditions and other potential confounders was undertaken by a linear regression adjusted for right censoring (Tobit regression), because no value could be above 100 but 312 of the values “piled up” at 100 [22].

The model includes age, gender and income as potential confounding variables, number of prescribed medications as a measure of the complexity of the overall health conditions, and years with current GP and number of visits to a GP in the last three months as factors which may also play a role in the degree of coordination.

Ethics approval for the survey was obtained from the Australian National University Human Research Ethics Committee (Protocol number: 2010/468) in 2010. All respondents provided informed consent to participate by returning completed questionnaires.

## Results

### Sample structure

Table 2 shows that sample responses varied across sub-samples, with more men responding within the NDSS (diabetes) sample but more women in the LFA and the NSA samples. While there was little difference in the average ages across the samples, the NDSS and LFA groups were on average older than those in the NSA population. Those in the samples with defined conditions were on average less well educated, of lower income (with lower income for LFA group than NDSS group), and of poorer health as reflected by the number of comorbid conditions or the number of medications taken regularly.

A total of 1,865 of the 2,540 potential respondents regularly consulted with two or more health care professionals and were asked who organised their health care. Table 3 shows that respondents in the NSA sample are more likely to manage their own care or report having no-one organise their care than those in the NDSS and LFA samples, while the condition based groups are more likely to have their care organised by health care professionals.

Significant differences exist in the number of GP attendances with those whose care is managed by a health care professional more likely to have attended a GP in the previous three months (mean 2.8; 95% CI 2.6–2.9), than those whose care is organised by themselves or a carer (2.2; 95% CI 2.1–2.3) or those whose care is managed by no-one (1.5; 95% CI 1.3–1.7).

Of the participants, 1,821 provided sufficient information for their coordination index score to be derived, and 1,614 of these also identified the person who organised their care.

Table 4 shows that overall those whose care is organised by a health professional have a significantly higher level of coordination than those who manage their own care, while the latter group reports a significantly higher level of coordination than those with no-one organising their care. Table 4 also shows that those in the NDSS sample have higher levels of the composite coordination index, regardless of who provided the coordination although few of the differences are significant.

If the main organiser is examined in more detail, when a partner/spouse is the main organiser the mean score is significantly higher than if the person manages their own care; and if a practice nurse is the main organiser the score is significantly higher than if a GP manages the care (although the practice nurse sample is quite small). The practice nurse score relative to the GP score is dominated by the question relating to care plans, with 90% of people whose main organiser is a practice nurse reporting having a care plan, compared to only 52% of those mainly organised by a GP. The difference between the partner/spouse managing and own management is dominated by similar questions to those which dominate in Table 5 – whether the doctor had information needed when you visited, whether you have a professional care plan, and whether the GP knew if another provider changed prescriptions.

Table 5 shows the mean value for each of the variables which contribute to the composite coordination index relative to the main organiser. Broadly speaking, the higher the level of organisation the more likely it is that a doctor would have the necessary information from another health care professional to hand, and the more likely that the patient would have a care plan.

While other differences in Table 5 are generally not significant due to the results for self-management and professional management being of a similar order, and due to the small sample for those with no organiser, a number of broad patterns are visible. Consistent with Table 4, in four of the seven components those with no organiser had the lowest score, and of the four, those with a professional organiser had the highest score. It is also noteworthy that none of the groups had a material number of people reporting a need to make another appointment due to the GP not having necessary information.

The relationships shown in Table 4 indicate that the degree of coordination is related to who mainly organises care. This is confirmed by the regression analysis shown below in Table 6. The regression shows that conditional on the other factors, having a health care professional manage your care leads to significantly higher levels of the composite coordination score. While only significant at 10%, self-management also shows higher levels of coordination than having no main organiser of care. The patients in the diabetes sample show significantly higher levels of coordination than those in other samples, and those who take more medications also have a higher level of coordination.

Those older than 60 years have higher levels of coordination than those under 60 years. Those with one or more visits to the GP in the last three months have significantly higher coordination levels than those with no visits, the effect increasing with number of visits. Gender and income have no significant effect on the care coordination index, however the period spent with the current GP had a significant positive effect.

## Discussion

The development of a composite coordination index based on actual activities related to care coordination allows analyses of care organisation across a range of factors; and can make useful distinctions between the degree of care when organised by the patient or by a health care professional.

The analyses in this study show that the groups we would expect and hope to have more coordination – people with more complex conditions as evidenced by their medications, older people, people with diabetes – indeed do have more coordination consistent with previous findings [23]. Those whose care is managed by a health care professional have better coordination, regardless of whether they are GPs or specialists but (acknowledging the small sample) there are indications that having a practice nurse as care manager may improve measured coordination, although this link must be taken as tentative at this stage.

Care coordination is one of four pillars for primary care [6, 24]. Bodenheimer et al. [25] suggest that one of the ten building blocks of high performing primary care is comprehensiveness and care coordination. They go on to suggest that improving care coordination requires teams that often include a care coordinator or referral coordinator whose sole responsibility is care coordination. Kmietowicz [27] also concludes, “The best schemes for improving the care of people with long term or chronic conditions are those that are led by GPs, backed by a team of other health and social care professionals with common objectives and who audit their performance” [p. 1].

The modelling in this study confirms that having a professional care coordinator provides the highest levels of coordination, and confirms the importance of continuity of care by the significant effect of the period a patient attended the same GP. It also suggests – if less strongly – that self or family/friend coordination leads to better coordinated care on these service based measures than when no-one is recognised by the individual as taking the coordination lead. This is consistent with what we know of patient activation in patient-centred care as proposed by Wagner's model [13]. It is also consistent with the views of Bodenheimer et al. [25] who suggested a “medical home” may be a model for improving coordination. A separate paper on medical homes suggested a shift is required from today's approach where: “Patients are responsible for coordinating their own care… [to a model where] a prepared team of professionals coordinates all patients’ care, where we track tests and consultations, and follow-up” [28, p. 6]. This is not to say patients with chronic diseases should not take a coordination role. Patients with chronic diseases should be encouraged to self-manage their condition and it is likely that this would include aspects of coordinating their care. Goodwin et al. [26] suggest:

a holistic focus that supports service users and carers to become more functional, independent and resilient, and to live well by managing their conditions in the home environment, is preferable to a purely clinical focus on managing or treating medical symptoms. (p. iv)

As the difference in level of coordination between “self or carer organises” and “health professional organises” is statistically significant in our study, the challenge for both patients and professionals is to work collaboratively to identify how care organisation is best managed, to individualise care plans and provide professional support accordingly.

The main factors reflecting differences between the groups were the availability of care plans, and the access by health care professionals to information from other providers. One significant effect across most measures was that those who felt that no-one organised their care were more likely to find notably poorer information flows. An opportunity to understand and improve the coordination of this group of patients who comprise almost 10% of the population using two or more health care professionals has the potential to improve the quality of care they receive. For example, in patients with heart failure and a management plan the time to the next preventable hospital admission is delayed [29].

A silver bullet solution often proposed to better support information flows is the availability of an electronic health record, which has been shown to support care coordination [30] and improve quality of care [31]. Goodwin et al. [26] also recommended:

there is a need for shared electronic health records to support the process, but a “high-touch, low-tech” approach has value in promoting face-to-face communication, fostering collaboration and enabling meaningful conversations about the needs of patients with complex needs. (p. iv)

## Limitations

The study explores care coordination from the patient's perspective using an index which was developed after the survey was undertaken. The index does not cover all dimensions of care co-ordination (see for example Shulz et al. [12] who identified 19 different domains of care coordination in 96 different scales), but was developed by an expert group and follows the logic of other measures which address important domains of care coordination, in this case mainly those relating to informational and management issues. As sample sizes in some categories can be small, some potentially material differences are not statistically significant particularly in relation to those who report no coordination of their care. The question on who coordinates care is purely from the patient's perspective, which may be different to the clinician's perspective. However, it provides a unique view through the lens of a patient on how they experience care coordination.

## Conclusion

When individuals report taking responsibility for the coordination of their care, measures of service coordination are higher than when no-one is recognised as coordinating care. However, care coordination by a health care professional is associated with indicators suggesting better coordination, and other literature suggest that this should be a team based approach led in general practice. There is a need for improved care coordination in Australia and it is necessary for other conditions (e.g. chronic pain, as well as, diabetes and cardiovascular disease). Our data suggest that in moving towards care coordination there are opportunities to improve the quality of the care coordination process itself, and the key enabler appears to be the availability of clinical information.

## Figures and Tables

**Table 1.**
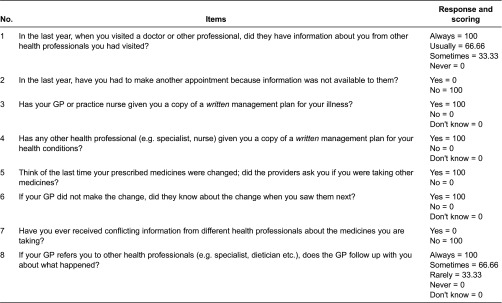
Questions and scoring used in composite coordination index

Note: In all cases “Not applicable” responses set to missing.

**Table 2.**
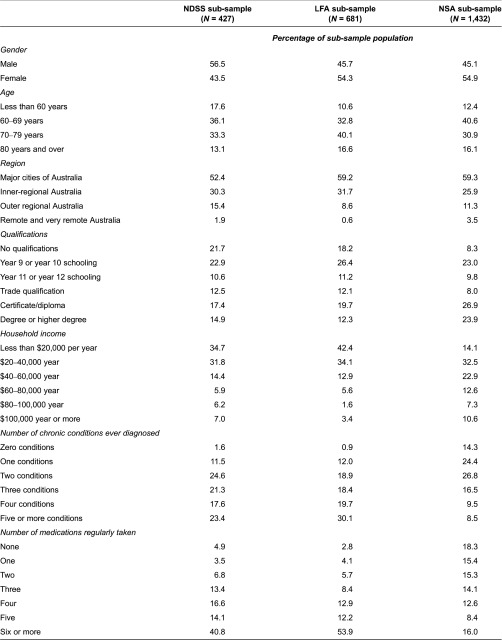
Socio-demographic and chronic disease characteristics of samples

**Table 3.**
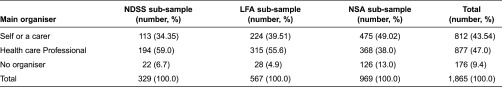
Main organiser of care in sub-samples

**Table 4.**
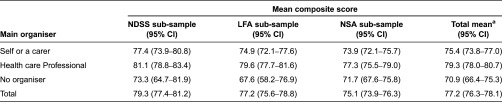
Mean composite coordination index by who organises care and sub-sample

^a^Total mean is the unweighted average across the three sub-samples, taken to avoid the NSA sample dominating results.

**Table 5.**
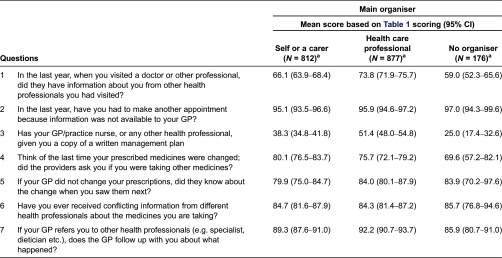
Components of composite coordination index

^a^Note that not all respondents answered all questions.

**Table 6.**
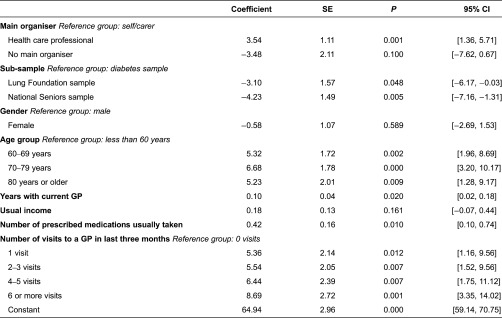
Tobit regression of the composite coordination index against main organiser and other factors (*N* = 1499)
